# Amputation of Hip-Joint and Excision

**Published:** 1880-04

**Authors:** 


### AMPUTATION OF HIP-JOINT AND EXCISION.

PROFESSOR LISTER, before the Clinical Society of London, in February, stated that he had never seen a case in which amputation of the hip was called for in preference to excision. Since seeing Professor Sayre illustrate his method at the Philadelphia Congress, he had followed that plan, which involved only a limited incision, did not require the ligature of any vessel, and gave free access to the joint, whilst the subperiostial section was of great advantage. The last case he had operated on was in a young lady twenty-five years of age, the subject of hip disease from early childhood. The limb was flexed and riddled with profusely discharging sinuses. He excised after Sayre's method, broke through the anchylosis, and found the acetabulum to be perforated. All the diseased bone being cleared away, chloride of zinc was applied, and now, three weeks after, there was far less discharge than at any time before the operation.

In regard to the treatment of abscesses in the hip, he would mention three cases he had had at King's College Hospital in adults. If treated by simple incision, frequent washing out, and free drainage, the result might have been different from what it was. One was a young woman in whom the inflammation was attributable to sleeping in a damp bed; the second was a man in whom it had followed acute rheumatism; and the third was in an old woman, also rheumatic in origin. In each case the abscess was opened and treated antiseptically; no fever followed, and no shortening of the limb. No one could imagine the satisfaction at such issues; for had these cases been treated in the ordinary way, probably all three patients would have died. In strumous cases—where shortening exists, with or without perceptible grating—he should open the abscess antiseptically, and often more useful limbs are thus preserved than by excision; and even in advanced cases, for it is surprising what may be done by repair. In one case he had at Edinburgh, treated on this plan, he removed much carious substance, and the case went on so well that he thought it would not come to excision. No pus was formed, and the patient was almost well, only a small sinus oozing serum remaining when the antiseptic dressings were given up. Suppuration followed, and excision had to be resorted to. He therefore urged strongly that if they wished to do full justice to their patients they ought to use antiseptic measures when the skin was unbroken. There should be no difference in their practice of hospital patients and private cases. If hospital cases were to be deprived of all the benefits they could bring to them, then hospital accommodation was shamefully and disgracefully inadequate.—*London Lancet*, *March 1880.*

